# Proteomic analysis of premature umbilical cord blood and its relationship with bronchopulmonary dysplasia

**DOI:** 10.1186/s13052-025-01926-8

**Published:** 2025-03-17

**Authors:** Jia Chen, Yuanye He, Ying Liu, Zhiwei Guo, Longli Yan, Xiaotao Jiang, Weiwei Gao

**Affiliations:** 1 National Key Clinical Specialty Construction Project/Neonatology Department, Guangdong Women and Children Hospital, Guangzhou, 510000 Guangdong China; 2 Guangdong Neonatal ICU Medical Quality Control Center, Guangzhou, 510000 Guangdong China; 3 Department of Laboratory Medicine, Guangdong Second Provincial General Hospital, Guangzhou, 510317 Guangdong China; 4 Center for Medical Research On Innovation and Translation, Institute of Clinical Medicine, School of Medicine, Guangzhou First People’s Hospital, South China University of Technology, Guangzhou, 510000 Guangdong China; 5 First College of Clinic Medicine, Guangzhou University of Chinese Medicine, Guangzhou, 510000 Guangdong China

**Keywords:** Bronchopulmonary dysplasia, Plasma proteomics, Preterm infants, Biomarkers

## Abstract

**Background:**

Bronchopulmonary dysplasia (BPD) frequently occurs in preterm infants, causing significantly impaired lung function and increased mortality rates. Studies on plasma protein levels can facilitate early detection of BPD, enabling prompt intervention and a decrease in mortality.

**Methods:**

We conducted a prospective observational study involving proteomic sequencing of plasma samples from 19 preterm infants. Our analysis included principal component analysis, volcano plots, heatmap analysis, enrichment analysis, and receiver operating characteristic (ROC) analysis.

**Results:**

Infants with BPD were characterized by increased levels of lipopolysaccharide (LPS)-binding protein (LBP), X-ray repair cross-complementing protein 6 (XRCC6), GLI pathogenesis-related 1 (GLIPR1), Golgi membrane Protein 1(GOLM1), immunoglobulin kappa variable (IGKV1-5), and immunoglobulin kappa variable 1–33 (IGKV1-33) in cord blood. Additionally, gene pathway analysis revealed a significant correlation between the pathways associated with these genes and BPD, particularly pathways involved in the immune system, innate immune system, neutrophil degranulation, prion diseases, regulation of the actin cytoskeleton, and the MAPK signaling. The proteins amine oxidase copper containing 3 (AOC3) and H4 clustered histone 6 (H4C6) were diagnostically significant. Additionally, H4C6 was negatively correlated with intraventricular haemorrhage and patent ductus arteriosus, and positively correlated with antenatal steroid administration. AOC3 was also positively correlated with antenatal steroid use.

**Conclusions:**

Our findings suggest that the development of BPD is associated with changes in the plasma proteome of preterm infants. Specifically, the levels of AOC3 and H4C6 in the bloodstream could serve as biomarkers for the early detection of BPD in preterm infants. Furthermore, we found that GOLM1, lipopolysaccharide (LPS)-binding protein, XRCC6, and the contribution of neutrophil degranulation may play a crucial role in the development of therapies for BPD.

## Background

Bronchopulmonary dysplasia (BPD) is a chronic lung disease in neonates, caused by prolonged use of mechanical ventilation (a machine that assists air movement in and out of the lungs) and/or long-term oxygen supplementation. It is the most common complication of preterm birth and affects both the alveoli and pulmonary vasculature , leading to severe short and long-term complications [1]. With significant improvements in perinatal and neonatal care, the survival rate of premature infants has increased over the past decades. In particular, the survival rate of preterm infants with BPD who receive prenatal steroids for lung maturation, surfactant therapy, and changes into gentle ventilation strategies is higher [2]. However, preterm infants remain at high risk for mortality due to a significant increase in late respiratory morbidity. Therefore, early identification and early treatment of premature infants at high risk of BPD are necessary. Currently, the clinical diagnosis of BPD is still based on clinical symptoms and lacks relevant detection measurable indicators [3]. A previous study revealed that some novel circulating biomarkers in preterm infants may improve early identification of at-risk preterm newborns and provide novel targets for future preclinical and clinical studies for disease prevention [4]. However, no specific clinical indicator that can predict the occurrence of BPD has been identified.

Proteomics involves the measurement of protein levels in accessible biofluids (for example, plasma, urine or cerebrospinal fluid) [5]; it also has the ability to identify the activity of multiple signalling pathways from very small amounts of blood [6]. Proteomics plays a vital role in increasing the understanding of disease processes, developing new biomarkers for more accurate disease diagnosis, stratifying disease risk, and expediting drug development.

Therefore, our main objectives, in this study was to first identify relevant differentially expressed proteins between preterm infants with BPD and controls via a large-scale proteomic strategy. We further sought to identify signalling pathways associated with the differentially expressed proteins. Finally, we analysed the correlations of these proteins, which may be associated with the diagnosis of BPD, with clinical information.

## Methods

### Study design and patient population

This study is a prospective observational study and all donors were enlisted from Guangdong Women and Children Hospital in China. The diagnosis of bronchopulmonary dysplasia (BPD) adhered to the standards proposed by Jensen et al. in 2019 [3], indicating that infants born at gestational ages less than 32 weeks require oxygen saturation to be maintained at 90% to 95% by 36 weeks [7]. The inclusion criteria comprised: (a) birth at our hospital, (b) gestational age <32 weeks and a weight < 1,500 g, (c) absence of sepsis, suppurative meningitis, pneumonia, and other serious infectious diseases, and (d) availability of complete clinical data with family consent, including signed informed consent. The exclusion criteria encompassed patients with concurrent serious lung diseases or pulmonary infections, congenital conditions (such as congenital heart disease, malformations, or metabolic disorders), anomalies affecting the central nervous, respiratory, or digestive systems, individuals with necrotizing enterocolitis or digestive tract perforation, those with chromosomal abnormalities or diaphragmatic hernia, as well as those who deceased or were automatically discharged before reaching 36 weeks of corrected gestational age.

### Data collection

In addition to clinical information, cord blood samples were collected from the infant after birth. The blood samples were collected into an ED TA-plasma tube and centrifuged at 2,000 rpm for ten minutes at normal atmospheric temperature. The plasma was subsequently stored at -80°C until sequencing.

### Proteomic analyses

Protein analyses were performed at the Beijing Genomics Institute Center. TimsTOF Pro was used to acquire mass spectrometry (MS) data for 19 samples in data-independent acquisition (DIA) mode. Quantification of peptides and proteins was performed with MSstats software packages. These analyses were performed via next-generation label-free quantitative proteomics technology, and the DIA analysis pipeline provided an ideal differential proteomic analysis and a proteomic quantification platform for large amounts of samples. We utilized the UniProt protein database, the protein database from NCBI Reference Sequence (RefSeq) protein database and databases from other sources. The DIA data were analysed using indexed retention time (iRT) peptides for retention time calibration. A false-positive quality control analysis was subsequently conducted with a false discovery rate (FDR) of 1% according to the target-decoy model applicable to SWATH-MS (SWATH-MS utilizes DIA for large-scale data collection and differential analysis through the MSstats package), thus yielding significant quantitative results. MSstats was used for the statistical evaluation of significant differences in proteins or peptides from differential samples. Proteins were considered differentially expressed if they met the criteria of fold change (FC) >1.1 and *P* value<0.05, which indicated significance. Moreover, enrichment analysis was performed on the differentially expressed proteins.

## Results

### Baseline clinical characteristics of the selected preterm infants

Nineteen preterm newborns were included in the study. A total of 19 plasma samples were collected for proteomic analysis. Nine infants who developed BPD during their hospitalization in the neonatal intensive care unit (NICU) formed the BPD group, and 10 infants who did not meet the diagnostic criteria for BPD formed the non-BPD group. The overall characteristics of the cohort and the differences in selected variables between the analysed groups are shown in Table 1. Infants who developed BPD during hospitalization were characterized by a longer total duration of ventilator use and total oxygen time. The baseline clinical characteristics of premature infants with BPD were as follows: female sex, 5 (55.6%%); mean birth weight, 1.20 kg; mean gestational age (GA), 28.87 weeks; mean maternal age, 31.56 years; caesarean, 6 (66.7%);prenatal steroids, 3 (33.3%); premature rupture of membranes (PROM) >18h, 3 (33.3%); gestational diabetes, 4 (44.4%); chorioamnionitis, 0 (0%); patent ductus arteriosus (PDA), 6(66.7%); early-onset sepsis, 0 (0%); intraventricular haemorrhage (IVH), 5 (55.6%); retinopathy of prematurity, 0 (0%); mean total duration of ventilator use, 1078.22h; mean total oxygen time, 1465.00 h; and mean pH of the first blood gas analysis, 7.25.

**Table 1:** Comparison of selected demographic variables and hospitalization data of the patients in the studied groups

Patient characteristics	BPD (*N* = 9)		No BPD (*N* = 10)		*P* value
	N	SD	N	SD	
Sex(female)	*n* = 5(55.6%)		*n* = 6(60%)		1.000
Birth Weight(kg), mean	1.20	0.28	1.21	0.20	0.920
GA weeks, mean	28.87	1.52	30.30	1.43	0.050
Maternal Age, mean	31.56	3.43	32.20	5.63	0.770
Caesarean	*n* = 6(66.7%)		*n* = 7(70%)		1.000
Prenatal steroid usage	*n* = 3(33.3%)		*n* = 7(70%)		0.179
PROM > 18 h	*n* = 3(33.3%)		*n* = 2(20%)		0.628
Gestational diabetes	*n* = 4(44.4%)		n = 3(30%)		0.650
Chorioamnionitis	n = 0(0%)		n = 0(0%)		
Apgar score (1 min), mean	7.89		7.20		0.497
Apgar score (5 min), mean	9.00		8.80		0.720
Complication					
Patent ductus arteriosus	*n* = 6(66.7%)		*n* = 3(30%)		0.179
Early onset sepsis	*n* = 0(0%)		*n* = 1(10%)		1.000
Intraventricular hemorrhage	*n* = 5(55.6%)		*n* = 2(20%)		0.170
Retinopathy of prematurity	*n* = 0(0%)		*n* = 0(0%)		
PH (the first blood gas analysis), mean	7.25	0.10	7.28	0.11	0.679
Total time of using the ventilator(h), mean	1078.22	713.10	287.00	114.62	0.004
Total oxygen time(h), mean	1465.00	687.82	358.20	102.23	0.000

### Proteomic analysis of premature infants with BPD and without BPD

**Fig. 1:**
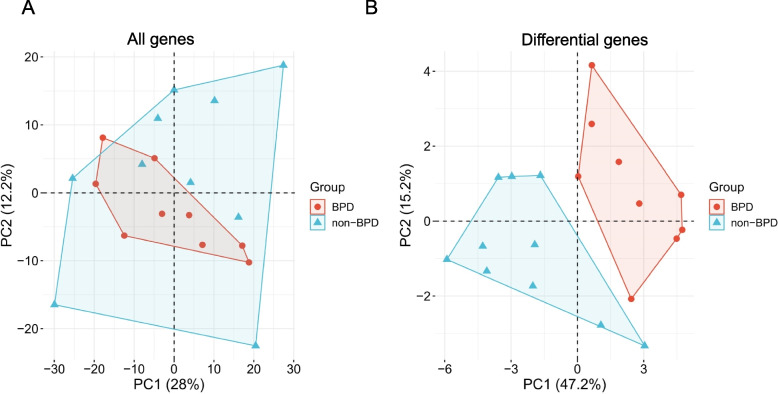
PCA (principal component analysis) score plot (**A**) Principal Component Analysis (PCA) Plot for All Genes in premature infants with BPD and without BPD; **B** Principal Component Analysis (PCA) Plot for Differentially Expressed Genes in premature infants with BPD and without BPD

Previous studies have indicated that early levels of angiogenic proteins are linked to a greater risk of bronchopulmonary dysplasia (BPD) in preterm infants. We reasoned that proteomic biomarkers may be used to identify infants at risk for BPD. To test this hypothesis, we adopted a large-scale proteomic strategy. Nine infants with BPD and 10 infants without BPD were included in the study. The scatter plots of the principal component analysis scores of all proteins and the differentially expressed proteins between the two groups of subjects are shown in Figure 1, respectively. Most samples in the two groups of samples were not fitted in most part, and the differentially expressed proteins of between premature infants with BPD and those without BPD were significantly different (Figure 1 B).

### Analysis of differentially expressed proteins in premature infants with BPD and without BPD

**Fig. 2:**
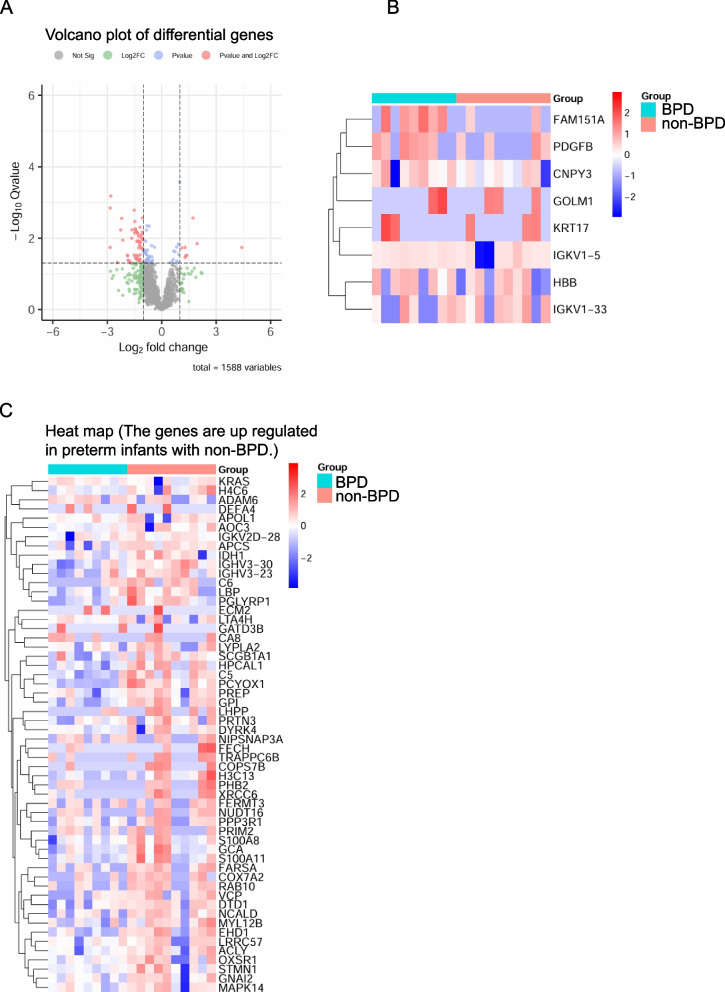
Differential proteins in premature infants with BPD and without BPD. **A** BPD vs. non-BPD Group Differential Genes Volcano Plot Analysis; **B** Heat maps of 8 different genes were analyzed between the BPD and non-BPD groups; **C** There were 74 proteins with the significant indicated significant differences in genes between BPD and no BPD

A volcano plot of differentially expressed proteins was generated using the R programming language to visualize the differences (Figure 2 A). Seventy-four proteins significantly differed (*P* <0.05) between BPD patients and non-BPD patients. In addition, Of the 74 proteins, 8 proteins were significantly upregulated in preterm infants with BPD, and 66 proteins were significantly upregulated in the control group (Figure 2; *P* <0.05; |log2FC|≤1). The proteins with the greatest FC values in preterm infants with BPD were LBP, X-ray repair cross-complementing protein 6 (XRCC6), glucose-6-phosphate isomerase (GPI), Golgi membrane protein 1 (GOLM1), immunoglobulin kappa variable 1-5 (IGKV1-5), and I immunoglobulin kappa variable 1-33 (GKV1-33) (Figure 2).

### Analysis of corresponding reaction pathways

**Fig. 3:**
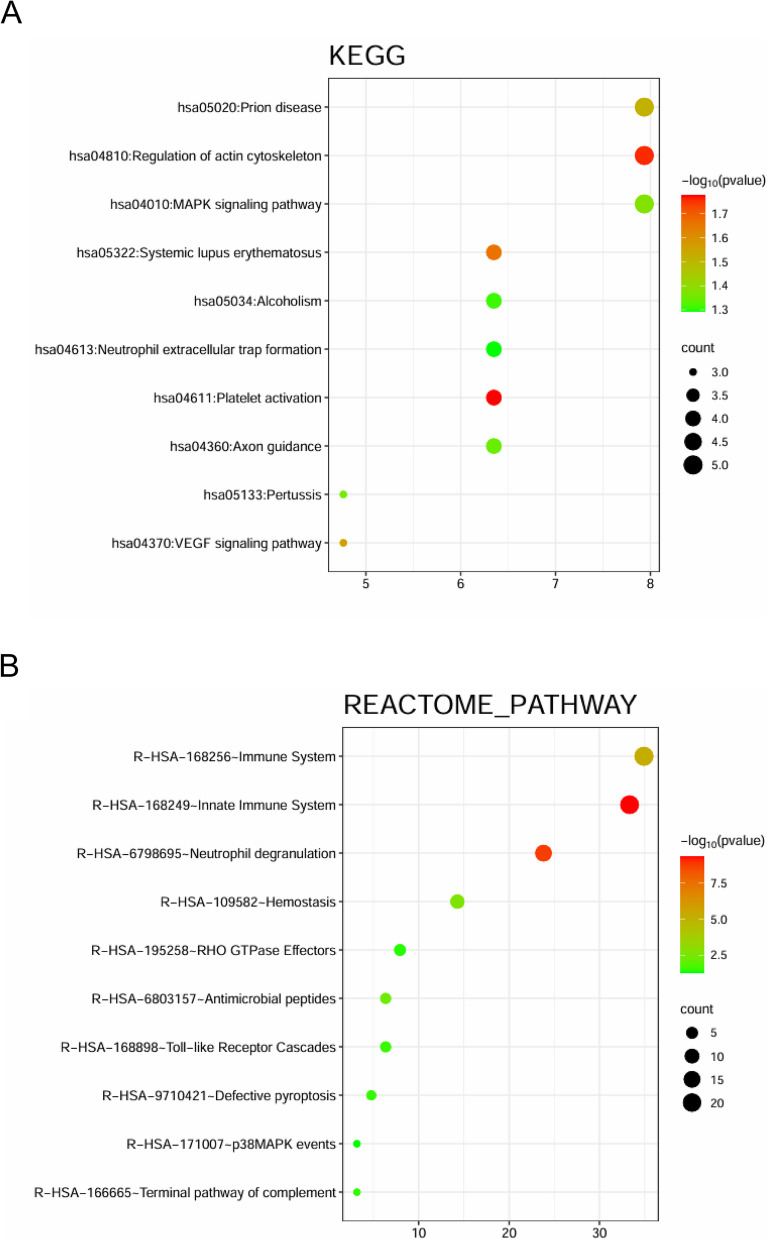
Differential proteins pathway enrichment maps of the premature infants with BPD and without BPD. **A** KEGG showed different signaling pathways between BPD and non-BPD groups; **B** Reactome analysis showed different signaling pathways between BPD and non-BPD groups

To elucidate the role of these differentially expressed proteins in BPD pathogenesis, the proteins were mapped to reaction pathways and KEGG pathways using their protein ID (Figure 3). The differentially expressed proteins were associated with the following pathways: innate immune system, neutrophil degranulation, immune system, haemostasis, antimicrobial peptides, platelet activation, regulation of actin cytoskeleton, systemic lupus erythematosus, Toll-like receptor cascades, VEGF signalling, prion disease, terminal pathway of complement, defective pyroptosis, GTPase effectors, MAPK signalling, pertussis, axon guidance, alcoholism, MAPK events, and scavenging of heme from plasma (Figure 3, *P* < 0.05). The most significant pathways in the development of BPD in preterm infants were immune system (*P* < 0.001, count: 21), innate immune system (*P* < 0.001, count: 15), neutrophil degranulation (*P* < 0.001, count: 22), prion disease (*P* < 0.05, count: 5), regulation of the actin cytoskeleton (*P* < 0.05, count: 5), and the MAPK signalling pathway (*P* < 0.05, count: 5).

### Receiver operating characteristic (ROC) curves of the differentially expressed proteins

**Fig. 4:**
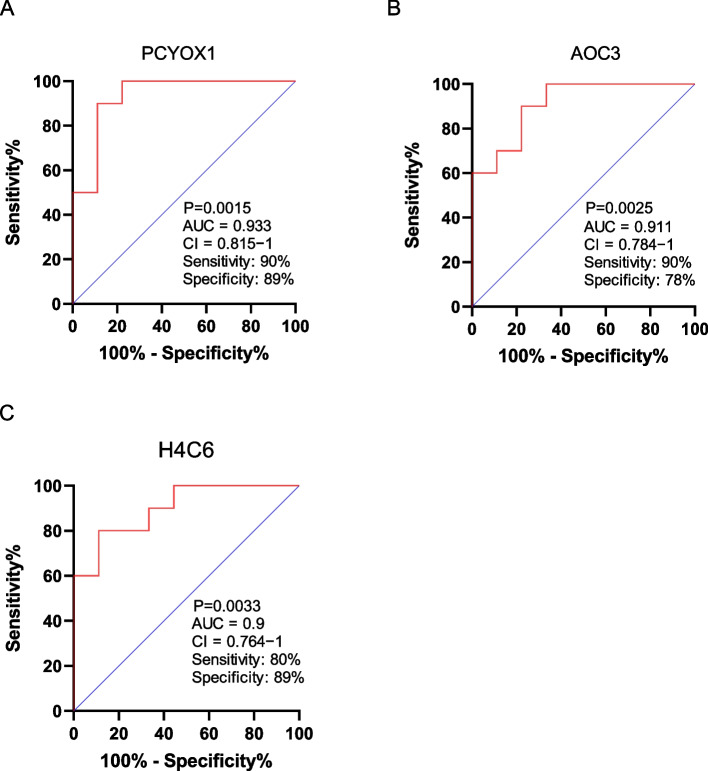
Receiver operating characteristic curve (ROC) of the differential proteins. **A** ROC diagnostic value analysis of PCYOX1; **B** ROC diagnostic value analysis of AOC3; (**C**) ROC diagnostic value analysis of H4C6

To validate the correlation between differentially expressed proteins and BPD, we generated ROC curves and calculated the areas under the curve (AUCs) to identify the proteins with diagnostic value for BPD. We found that the AUCs of prenylcysteine oxidase 1 (PCYOX1), amine oxidase copper containing 3 (AOC3) and H4 histone clustered 6 (H4C6) were greater than 0.9 (Figure 4), and all the proteins had high specificity and sensitivity, suggesting diagnostic capabilities.

### Correlation analysis investigating the relationships between proteins with diagnostic value for BPD and clinical indicators

**Fig. 5:**
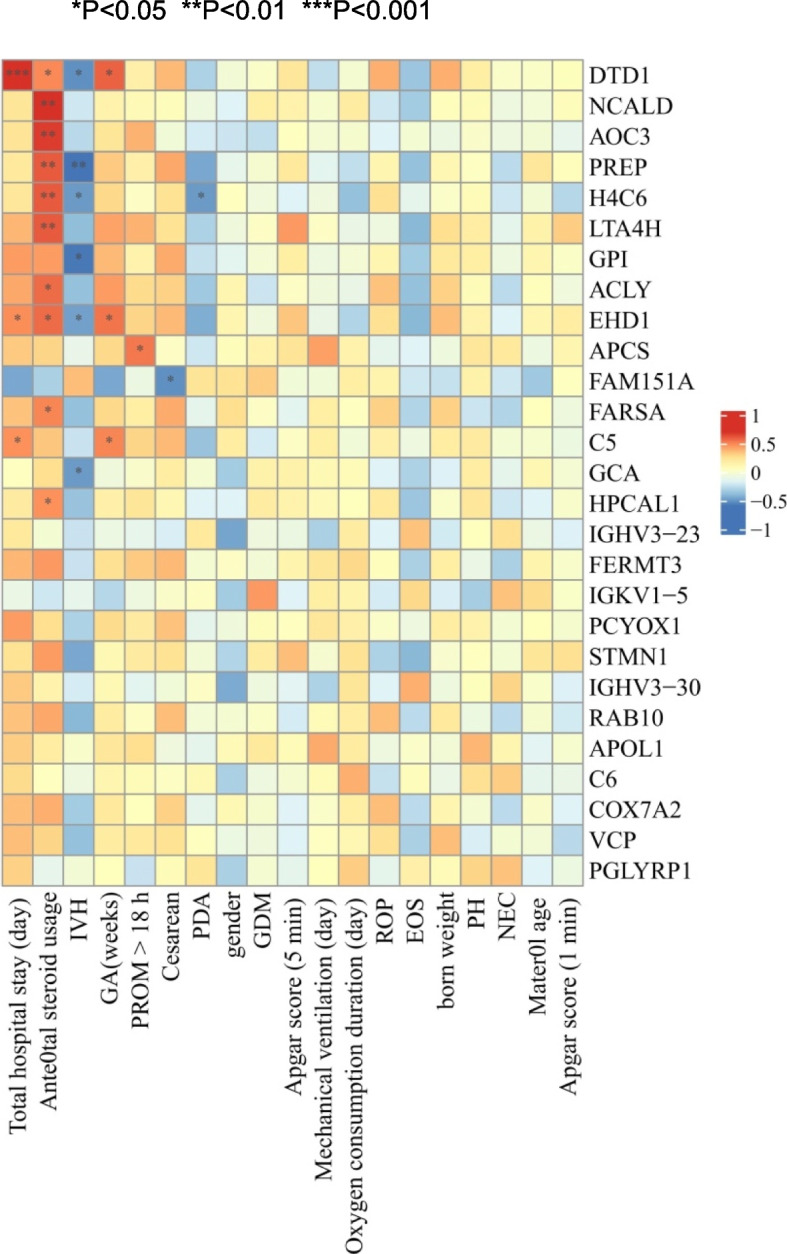
A correlation analysis was conducted to investigate the relationship between proteins capable of diagnosing BPD and clinical indicators

We further analysed the correlations of these proteins that could be used to diagnose BPD with clinical information (Figure 5). H4C6 was negatively correlated with IVH (correlation index: -0.494, *P* value < 0.05) and PDA (correlation index: -0.494, *P* value < 0.05) but positively correlated with antenatal steroid usage (correlation index: -0.613, *P* value < 0.01). AOC3 was also positively correlated with antenatal steroid usage (correlation index: -0.681, *P* value < 0.01). PCYOX1 was weakly correlated with clinical information. Decreased levels of H4C6 in umbilical cord blood may be associated with an increased risk of IVH or PDA in patients with BPD. The prenatal use of corticosteroids may be related to increased levels of H4C6 and AOC3 in infants with BPD.

## Discussion

BPD is a common consequence of prematurity, often exacerbating lung damage and increasing mortality rates. Consequently, early intervention and treatment are imperative for premature infants at risk of BPD. A systematic review identified several clinical and laboratory biomarkers that may prove valuable in the early detection and treatment of infants at increased risk of developing BPD [8]. In a recent study, Bose et al. reported a strong correlation between increased blood concentrations of various proinflammatory cytokines, adhesion molecules, and proteases and the onset of BPD [9]. These findings suggest that inflammation-associated proteins in neonatal blood could function as biomarkers for modifiable biological processes implicated in the pathogenesis of BPD. Understanding the impact of differentially expressed proteins on the development of BPD, particularly proteins crucial for normal lung development, is essential for leveraging lung proteomics in the prevention and treatment of BPD. However, systematic research on the protein characteristics of premature infants with BPD is still lacking. If these characteristics can be systematically determined, early diagnosis, treatment, and intervention of BPD will improve. Proteomics is currently widely applied in studies aimed at predicting the risk of infectious diseases in neonates, such as sepsis and chorioamnionitis [10, 11]. In summary, we employed advanced proteomic technologies, which are currently capable of profiling the circulating blood proteome in large population studies, to analyse 9 plasma samples from premature infants with BPD and 10 plasma samples from control groups infants.

Previously, in a proteomics study of 102 premature infants with and without BPD, Sanne Arjaans et al. reported a significant association between early increased levels of BMP10 and the subsequent increased risk of BPD and pulmonary hypertension. They suggested that proteomic biomarkers detected within the first week of life could identify infants at risk of developing BPD and/or pulmonary hypertension [4]. Our research group conducted plasma proteomics analyses on premature infants with and without BPD, identified differentially expressed proteins, and analysed the potential protein pathways related to the pathogenesis of BPD.

In this study, we first identified the proteins that significantly differed between infants with BPD and those without BPD. Second, we identified the differentially expressed proteins, such as GOLM1, IGKV1-5, and IGKV1-33, that significantly increased in premature infants with BPD. According to the literature, GOLM1 can upregulate NEAT1 to promote pulmonary fibrosis, which is a major characteristic of BPD, before the implementation of antenatal steroids and surfactant therapy [12]. Pulmonary fibrosis after new treatment is characterized by less fibrosis [13], and GOLM1 may be useful for monitoring disease processes and the response to therapy. In addition, we found that LBP, XRCC6 and GPI increased significantly in premature infants with BPD. Lung inflammation is a hallmark of BPD. LBP is the main component of the outer membrane of gram-negative bacteria and its role in infections and inflammatory diseases has been widely studied [14]. The lung phenotype of mice exposed to early postnatal LPS was similar to that of humans with BPD [15]. Shrestha et al. reported that chronic LPS exposure plays a crucial role in lung development [16].

These findings are congruent with our findings, suggesting that LBP has significant value in the development of therapeutic targets for BPD in infants. Past studies suggest that pulmonary hypertension is associated with the postnatal course and outcomes of extremely premature infants, especially infants with severe BPD [17, 18]. XRCC6 (ku70) is a direct target of the newly identified PIM1, and a study has demonstrated that PIM1 phosphorylates KU70 and initiates DNA repair signalling in pulmonary artery smooth muscle cells (PAH-PASMCs) in pulmonary arterial hypertension (PAH) rat models; therefore, PIM1 inhibitors represent a therapeutic option for patients with PAH [19].

However, the pathogenesis of pulmonary hypertension in infants with BPD is not yet completely clear. A few studies have reported that XRCC6 is involved in BPD pathogenesis in preterm infants. This finding provides insight for researchers investigating the pathogenesis of BPD, suggesting that XRCC6 may play a role in the disease's development in preterm infants. GPI is an important enzyme in the glycolytic pathway. In high-carbon dioxide conditions, such as those in patients with chronic obstructive pulmonary disease, asthma, cystic fibrosis, BPD, and muscular dystrophies, organs and tissues are required to produce sufficient ATP through glycolysis [20]; our finding that GPI levels are significantly increased in premature infants with BPD supports the findings of this study. IGKV1-5 and IGKV1-33 are specific differentially expressed proteins identified in this study, but they have not been reported in clinical diseases. We speculate that these proteins may play a role in the development of BPD, but further research and confirmation are needed.

The protein pathway enrichment diagram fully reflects the pathway characteristics of differentially expressed proteins in the plasma of premature infants at a given time. The most prominent protein pathways in premature infants with BPD are the immune system, innate immune system, neutrophil degranulation, prion disease, regulation of the actin cytoskeleton and MAPK signalling pathways. A previous study revealed that immune system regulation was affected in a murine experimental model of BPD [21]. Dharmesh et al. confirmed that the macrophage-driven inflammatory interleukin (IL)-6/signal transducer and activator of transcription 3 (STAT3) response is present in the lungs or in the plasma of infants with BPD in a recent study [22]. These findings and our findings revealed that elevated increased plasma levels of immune system-related plasma cytokines could be as potential biomarkers to identify infants receiving oxygen who are at increased risk of developing BPD. Moreover, numerous studies have shown that innate immune system plays a role in the development of BPD; for example, type 3 innate lymphoid cells and type 2 innate lymphoid cells have been shown to regulate hyperoxia-induced lung injury via related pathways [23–26]. Furthermore, the overall consensus in the literature concerning the effect of hypoxia on neutrophil degranulation is that hypoxia augments granule exocytosis [27–29]. However, the role of neutrophil degranulation in the hypoxic environment is not clear, Some studies have shown that neutrophil degranulation has a protective effect on the host [30], however, it has recently been demonstrated that excessive release of neutrophil granules aggravates the development of chronic hypoxic lung disease by damaging tissue [30–32]. Therefore, the impact of neutrophil degranulation on development and its potential therapeutic significance in preterm infants with BPD should be explored. Chen et al. conducted epigenetic research on hyperoxia-exposed newborn rat lungs and reported that deregulation of the actin cytoskeleton pathway in lung tissues may be involved in the pathophysiology of hyperoxia-induced arrested alveolarization [33]. In addition, the current study confirmed that the MAPK signalling pathway is involved in the development of BPD, and the long noncoding RNAs (lncRNAs) H19 and PAR2 were identified as potential targets for the treatment of BPD [34, 35]. Prion disease is involved in the pathogenesis of several neurodegenerative diseases, but there are no studies related to its involvement in BPD.

AUC analyses revealed that PCYOX1, AOC3 and H4C6 could have diagnostic potential for premature infants with BPD. Correlation analysis was performed between differentially expressed proteins and clinical test indices and revealed that the change in proteins was correlated with the change in some clinical indices. H4C6 and AOC3 were significantly expressed in BPD infants; H4C6 was negatively correlated with IVH and PDA, and H4C6 and AOC3 were both positively correlated with antenatal steroids use. IVH is the most frequent and severe neurologic complication in premature infants [36]. Extremely preterm infants born at a GA of less than 28 weeks face the highest risk of developing PDA and its associated complications [37]. A previous study demonstrated that persistent PDA may result in adverse outcomes, including BPD [38]. In summary, IVH and PDA are both severe complications in preterm infants and may be associated with the development of BPD. Based on our proteomic analysis, we propose that the early detection of decreased H4C6 levels in the umbilical cord blood of preterm infants could serve as a potential diagnostic marker for coexisting IVH or PDA. The detection of this biomarker may enable early intervention in preterm infants, thereby reducing the occurrence of adverse outcomes. The administration of antenatal steroids, which are widely utilized to decrease the occurrence of complications and morbidities linked to preterm birth, has demonstrated effectiveness in reducing neonatal complications and mortality in previous trials [39]. However, the immunological mechanisms by which prenatal corticosteroid use reduces complications in preterm infants remain unclear [40]. Vascular adhesion protein-1 (VAP-1), also known as AOC3, is a versatile proinflammatory molecule with adhesive and enzymatic properties. The role of VAP-1 in inflammation, cardiovascular diseases, and tumour angiogenesis has been extensively studied, and it is considered a potential biomarker for disease diagnosis and prognosis [41]. H4C6 has no introns and encodes a replication-dependent histone that is a member of the histone H4 family. Currently, research on diseases associated with this gene is limited. On the basis of this analysis, we hypothesize that prenatal corticosteroid administration may reduce the incidence of BPD and its associated complications by modulating immune mechanisms involving H4C6 or AOC3. These findings demonstrate that AOC3 and H4C6 could be novel circulating biomarkers for early diagnosis of preterm infants with BPD. Furthermore, our findings may offer insights for researchers studying complications in preterm infants, suggesting that H4C6 and AOC3 could be associated with a reduction in the incidence of complications following prenatal corticosteroid administration.

This study has several limitations. The number of included infants was small, and it is likely that we missed important associations because of the lack of statistical power. In summary, our study has several implications for researchers interested in BPD. First, our findings suggest the possibility that two differentially expressed proteins in neonatal blood could serve as biomarkers for early diagnosis in premature infants with BPD. Second, some differentially expressed proteins, such as GOLM1, could be considered as indicators of the effect of BPD therapy. Finally, we identify several potential protein pathways that could offer some innovative targets for the intervention and treatment of premature infants with BPD.

## Conclusions

Our study revealed that AOC3 and H4C6 in the bloodstream of newborns could serve as biomarkers for the early detection of BPD in preterm infants. Identifying these biomarkers early could help identify at-risk preterm newborns and provide novel targets for future preclinical and clinical studies aimed at disease prevention. Additionally, the detection of H4C6 in the blood of infants with BPD may indicate the presence of complications such as IVH or PDA, providing clinicians with valuable information to tailor individualized treatment for BPD patients. The finding that increased levels of H4C6 and AOC3 in umbilical cord blood are associated with prenatal corticosteroid use in preterm mothers offers new genetic targets for researchers studying the mechanisms by which corticosteroids mitigate BPD and reduce complications. These findings could also provide novel strategies for the targeted prevention and treatment of BPD in preterm infants. Furthermore, our research indicates potential associations between specific proteins and protein pathways and possible interventions for BPD in preterm infants, which could inform the future development of individualized therapies. GOLM1 may serve as a useful biomarker for monitoring disease progression and therapeutic response. LBP, lncRNA H19, PAR2 and XRCC6 have significant implications for the development of therapeutic targets for BPD in infants. Neutrophil degranulation has an impact on the development of BPD in preterm infants and holds potential therapeutic significance.

## Data Availability

All data associated with this study are present in the paper or the Supplementary Materials.

## Abbreviations

BPD: Bronchopulmonary dysplasia
ROC: Receiver operating characteristic
MS: Mass spectrometry
DIA: Data Independent Acquisition
RefSeq: Reference Sequence
AUC: Area under the curve
IVH: Intraventricular hemorrhage
PDA: Patent ductus arteriosus
LPS: Lipopolysaccharide
LBP: Lipopolysaccharide-binding protein
GPI: Glucose-6-phosphate isomerase
STAT3: Signal transducer and activator of transcription 3

## References

[1] Thébaud B, Goss KN, Laughon M, Whitsett JA, Abman SH, Steinhorn RH, Aschner JL, Davis PG, McGrath-Morrow SA, Soll RF, Jobe AH. Bronchopulmonary Dysplasia Nat Rev Dis Primers. 2019;5(1):78. 10.1038/s41572-019-0127-7. 31727986 10.1038/s41572-019-0127-7 PMC6986462

[2] Trends in Care Practices, Morbidity, and Mortality of Extremely Preterm Neonates, 1993–2012 - PubMed. https://pubmed.ncbi.nlm.nih.gov/26348753/ (accessed 2024–03–14). 10.1001/jama.2015.10244 PMC4787615 26348753

[3] Jensen, E. A.; Dysart, K.; Gantz, M. G.; McDonald, S.; Bamat, N. A.; Keszler, M.; Kirpalani, H.; Laughon, M. M.; Poindexter, B. B.; Duncan, A. F.; Yoder, B. A.; Eichenwald, E. C.; DeMauro, S. B. The Diagnosis of Bronchopulmonary Dysplasia in Very Preterm Infants. An Evidence-Based Approach. Am J Respir Crit Care Med 2019, 200 (6), 751–759. 10.1164/rccm.201812-2348OC. 10.1164/rccm.201812-2348OC PMC6775872 30995069

[4] Arjaans S, Wagner BD, Mourani PM, Mandell EW, Poindexter BB, Berger RMF, Abman SH. Early Angiogenic Proteins Associated with High Risk for Bronchopulmonary Dysplasia and Pulmonary Hypertension in Preterm Infants. Am J Physiol Lung Cell Mol Physiol. 2020;318(4):L644–54. 10.1152/ajplung.00131.2019. 31967847 10.1152/ajplung.00131.2019 PMC7191476

[5] Dayon L, Cominetti O, Affolter M. Proteomics of Human Biological Fluids for Biomarker Discoveries: Technical Advances and Recent Applications. Expert Rev Proteomics. 2022;19(2):131–51. 10.1080/14789450.2022.2070477. 35466824 10.1080/14789450.2022.2070477

[6] Liumbruno G, D’Alessandro A, Grazzini G, Zolla L. Blood-Related Proteomics. J Proteomics. 2010;73(3):483–507. 10.1016/j.jprot.2009.06.010. 19567275 10.1016/j.jprot.2009.06.010

[7] The Diagnosis of Bronchopulmonary Dysplasia in Very Preterm Infants. An Evidence-based Approach - PubMed. https://pubmed.ncbi.nlm.nih.gov/30995069/ (accessed 2024–03–29). 10.1164/rccm.201812-2348OC PMC6775872 30995069

[8] Lal CV, Ambalavanan N. Biomarkers, Early Diagnosis, and Clinical Predictors of Bronchopulmonary Dysplasia. Clin Perinatol. 2015;42(4):739–54. 10.1016/j.clp.2015.08.004. 26593076 10.1016/j.clp.2015.08.004 PMC4662054

[9] Blood Protein Concentrations in the First Two Postnatal Weeks That Predict Bronchopulmonary Dysplasia among Infants Born before the 28th Week of Gestation. Pediatr. Res. 2011, 69 (4), 347–353. 10.1203/PDR.0b013e31820a58f3. 10.1203/PDR.0b013e31820a58f3 PMC3083822 21150694

[10] Fanos V, Stronati M, Gazzolo D, Corsello G. Metabolomics in the Diagnosis of Sepsis. Ital J Pediatr. 2014;40(1):A11. 10.1186/1824-7288-40-S1-A11.

[11] Fattuoni C, Pietrasanta C, Pugni L, Ronchi A, Palmas F, Barberini L, Dessì A, Pintus R, Fanos V, Noto A, Mosca F. Urinary Metabolomic Analysis to Identify Preterm Neonates Exposed to Histological Chorioamnionitis: A Pilot Study. PLoS ONE. 2017;12(12): e0189120. 10.1371/journal.pone.0189120. 29211784 10.1371/journal.pone.0189120 PMC5718427

[12] Wang Y, Hu D, Wan L, Yang S, Liu S, Wang Z, Li J, Li J, Zheng Z, Cheng C, Wang Y, Wang H, Tian X, Chen W, Li S, Zhang J, Zha X, Chen J, Zhang H, Xu K-F. GOLM1 Promotes Pulmonary Fibrosis through Upregulation of NEAT1. Am J Respir Cell Mol Biol. 2024;70(3):178–92. 10.1165/rcmb.2023-0151OC. 38029327 10.1165/rcmb.2023-0151OC

[13] Hwang JS, Rehan VK. Recent Advances in Bronchopulmonary Dysplasia: Pathophysiology, Prevention, and Treatment. Lung. 2018;196(2):129–38. 10.1007/s00408-018-0084-z. 29374791 10.1007/s00408-018-0084-z PMC5856637

[14] Meng L, Song Z, Liu A, Dahmen U, Yang X, Fang H. Effects of Lipopolysaccharide-Binding Protein (LBP) Single Nucleotide Polymorphism (SNP) in Infections, Inflammatory Diseases. Metabolic Disorders and Cancers Front Immunol. 2021;12: 681810. 10.3389/fimmu.2021.681810. 34295331 10.3389/fimmu.2021.681810 PMC8290185

[15] Shrestha AK, Bettini ML, Menon RT, Gopal VYN, Huang S, Edwards DP, Pammi M, Barrios R, Shivanna B. Consequences of Early Postnatal Lipopolysaccharide Exposure on Developing Lungs in Mice. Am J Physiol-lung C. 2019;316(1):L229–44. 10.1152/ajplung.00560.2017. 10.1152/ajplung.00560.2017 PMC6383495 30307313

[16] Shrestha AK, Menon RT, Yallampalli C, Barrios R, Shivanna B. Adrenomedullin Deficiency Potentiates Lipopolysaccharide-Induced Experimental Bronchopulmonary Dysplasia in Neonatal Mice. Am J Pathol. 2021;191(12):2080–90. 10.1016/j.ajpath.2021.09.001. 34508690 10.1016/j.ajpath.2021.09.001 PMC8647431

[17] Bhat R, Salas AA, Foster C, Carlo WA, Ambalavanan N. Prospective Analysis of Pulmonary Hypertension in Extremely Low Birth Weight Infants. Pediatrics. 2012;129(3):e682–9. 10.1542/peds.2011-1827. 22311993 10.1542/peds.2011-1827 PMC3289526

[18] Arjaans S, Zwart EAH, Ploegstra M, Bos AF, Kooi EMW, Hillege HL, Berger RMF. Identification of Gaps in the Current Knowledge on Pulmonary Hypertension in Extremely Preterm Infants: A Systematic Review and Meta-Analysis. Paediatr Perinat Epidemiol. 2018;32(3):258–67. 10.1111/ppe.12444. 29341209 10.1111/ppe.12444

[19] PIM1 (Moloney Murine Leukemia Provirus Integration Site) Inhibition Decreases the Nonhomologous End-Joining DNA Damage Repair Signaling Pathway in Pulmonary Hypertension. 10.1161/ATVBAHA.119.313763. 10.1161/ATVBAHA.119.313763 31969012

[20] Vohwinkel CU, Lecuona E, Sun H, Sommer N, Vadász I, Chandel NS, Sznajder JI. Elevated CO2 Levels Cause Mitochondrial Dysfunction and Impair Cell Proliferation. J Biol Chem. 2011;286(43):37067–76. 10.1074/jbc.M111.290056. 21903582 10.1074/jbc.M111.290056 PMC3199454

[21] Revhaug C, Bik-Multanowski M, Zasada M, Rognlien AGW, Günther C-C, Ksiązek T, Madetko-Talowska A, Szewczyk K, Grabowska A, Kwinta P, Pietrzyk JJ, Baumbusch LO, Saugstad OD. Immune System Regulation Affected by a Murine Experimental Model of Bronchopulmonary Dysplasia: Genomic and Epigenetic Findings. Neonatology. 2019;116(3):269–77. 10.1159/000501461. 31454811 10.1159/000501461

[22] Hirani, D.; Alvira, C. M.; Danopoulos, S.; Milla, C.; Donato, M.; Tian, L.; Mohr, J.; Dinger, K.; Vohlen, C.; Selle, J.; V. Koningsbruggen-Rietschel, S.; Barbarino, V.; Pallasch, C.; Rose-John, S.; Odenthal, M.; Pryhuber, G. S.; Mansouri, S.; Savai, R.; Seeger, W.; Khatri, P.; Al Alam, D.; Dötsch, J.; Alejandre Alcazar, M. A. Macrophage-Derived IL-6 Trans-Signalling as a Novel Target in the Pathogenesis of Bronchopulmonary Dysplasia. Eur. Respir. J. 2022, 59 (2), 2002248. 10.1183/13993003.02248-2020. 10.1183/13993003.02248-2020 PMC8850688 34446466

[23] Yao H, Zhu Y, Lu H, Ju H, Xu S, Qiao Y, Wei S. Type 2 Innate Lymphoid Cell-Derived Amphiregulin Regulates Type II Alveolar Epithelial Cell Transdifferentiation in a Mouse Model of Bronchopulmonary Dysplasia. Int Immunopharmacol. 2023;122: 110672. 10.1016/j.intimp.2023.110672. 37480752 10.1016/j.intimp.2023.110672

[24] Wang Q-W, Zhu Y, Wang Q-X, Lu H-Y. Changes and significance of type 2 innate lymphoid cells and their related factors in bronchopulmonary dysplasia. Zhongguo Dang Dai Er Ke Za Zhi. 2023;25(2):179–85. 10.7499/j.issn.1008-8830.2210005. 36854695 10.7499/j.issn.1008-8830.2210005 PMC9979383

[25] Cai J, Lu H, Su Z, Mi L, Xu S, Xue Z. Dynamic Changes of NCR- Type 3 Innate Lymphoid Cells and Their Role in Mice with Bronchopulmonary Dysplasia. Inflammation. 2022;45(2):497–508. 10.1007/s10753-021-01543-7. 35122179 10.1007/s10753-021-01543-7 PMC8956536

[26] Zhu Y, Mi L, Lu H, Ju H, Hao X, Xu S. ILC2 Regulates Hyperoxia-Induced Lung Injury via an Enhanced Th17 Cell Response in the BPD Mouse Model. BMC Pulm Med. 2023;23(1):188. 10.1186/s12890-023-02474-9. 37254088 10.1186/s12890-023-02474-9 PMC10230686

[27] Inflammation and bronchopulmonary dysplasia: a continuing story - PubMed. https://pubmed.ncbi.nlm.nih.gov/16702036/ (accessed 2024–03–24). 10.1016/j.siny.2006.03.004 16702036

[28] Genschmer KR, Russell DW, Lal C, Szul T, Bratcher PE, Noerager BD, Abdul Roda M, Xu X, Rezonzew G, Viera L, Dobosh BS, Margaroli C, Abdalla TH, King RW, McNicholas CM, Wells JM, Dransfield MT, Tirouvanziam R, Gaggar A, Blalock JE. Activated PMN Exosomes: Pathogenic Entities Causing Matrix Destruction and Disease in the Lung. Cell. 2019;176(1–2):113-126.e15. 10.1016/j.cell.2018.12.002. 30633902 10.1016/j.cell.2018.12.002 PMC6368091

[29] Tamura DY, Moore EE, Partrick DA, Johnson JL, Offner PJ, Silliman CC. Acute Hypoxemia in Humans Enhances the Neutrophil Inflammatory Response. Shock. 2002;17(4):269–73. 10.1097/00024382-200204000-00005. 11954825 10.1097/00024382-200204000-00005

[30] Lodge KM, Cowburn AS, Li W, Condliffe AM. The Impact of Hypoxia on Neutrophil Degranulation and Consequences for the Host. Int J Mol Sci. 2020;21(4):1183. 10.3390/ijms21041183. 32053993 10.3390/ijms21041183 PMC7072819

[31] Chalmers JD, Moffitt KL, Suarez-Cuartin G, Sibila O, Finch S, Furrie E, Dicker A, Wrobel K, Elborn JS, Walker B, Martin SL, Marshall SE, Huang JT-J, Fardon TC. Neutrophil Elastase Activity Is Associated with Exacerbations and Lung Function Decline in Bronchiectasis. Am J Respir Crit Care Med. 2017;195(10):1384–93. 10.1164/rccm.201605-1027OC. 27911604 10.1164/rccm.201605-1027OC PMC5443898

[32] Hypoxia upregulates neutrophil degranulation and potential for tissue injury - PubMed. https://pubmed.ncbi.nlm.nih.gov/27581620/ (accessed 2024–03–24). 10.1136/thoraxjnl-2015-207604 PMC5099189 27581620

[33] Chen C-M, Liu Y-C, Chen Y-J, Chou H-C. Genome-Wide Analysis of DNA Methylation in Hyperoxia-Exposed Newborn Rat Lung. Lung. 2017;195(5):661–9. 10.1007/s00408-017-0036-z. 28689251 10.1007/s00408-017-0036-z

[34] Mo W, Li Y, Chang W, Luo Y, Mai B, Zhou J. The Role of LncRNA H19 in MAPK Signaling Pathway Implicated in the Progression of Bronchopulmonary Dysplasia. Cell Transplant. 2020;29:096368972091829. 10.1177/0963689720918294. 10.1177/0963689720918294 PMC7289048 32308025

[35] PAR2 Overexpression is Involved in the Occurrence of Hyperoxygen-Induced Bronchopulmonary Dysplasia in Rats - PubMed. https://pubmed.ncbi.nlm.nih.gov/36657618/ (accessed 2024–03–24). 10.1080/15513815.2023.2166799 36657618

[36] Intraventricular hemorrhage in preterm babies - PMC. https://www.ncbi.nlm.nih.gov/pmc/articles/PMC7536465/ (accessed 2024–03–24).

[37] Therapeutic strategy of patent ductus arteriosus in extremely preterm infants - PubMed. https://pubmed.ncbi.nlm.nih.gov/31740267/ (accessed 2024–03–24). 10.1016/j.pedneo.2019.10.002 31740267

[38] Benitz WE. Treatment of Persistent Patent Ductus Arteriosus in Preterm Infants: Time to Accept the Null Hypothesis? J Perinatol. 2010;30(4):241–52. 10.1038/jp.2010.3. 20182439 10.1038/jp.2010.3

[39] Liggins GC, Howie RN. A Controlled Trial of Antepartum Glucocorticoid Treatment for Prevention of the Respiratory Distress Syndrome in Premature Infants. Pediatrics. 1972;50(4):515–25. 4561295

[40] Cole TJ, Short KL, Hooper SB. The Science of Steroids. Semin Fetal Neonatal Med. 2019;24(3):170–5. 10.1016/j.siny.2019.05.005. 31147162 10.1016/j.siny.2019.05.005

[41] M, D.; Rc, T.; Lm, Q.; Bk, T. Vascular Adhesion Protein-1 (VAP-1) in Vascular Inflammatory Diseases. VASA. Zeitschrift fur Gefasskrankheiten 2022, 51 (6). 10.1024/0301-1526/a001031. 10.1024/0301-1526/a001031 36200383

